# Systematic assessment of streptozotocin-induced diabetic metabolic alterations in rats using metabolomics

**DOI:** 10.3389/fendo.2023.1107162

**Published:** 2023-01-24

**Authors:** Qingying Si, Jinxiu Guo, Xiumei Yang, Yujin Guo, Linlin Wu, Dadi Xie, Pei Jiang

**Affiliations:** ^1^ Department of Endocrinology, Tengzhou Central People’s Hospital, Tengzhou, China; ^2^ Translational Pharmaceutical Laboratory, Jining First People’s Hospital, Shandong First Medical University, Jining, China; ^3^ Office of Scientific Research Management, Tengzhou Central People’s Hospital, Tengzhou, China; ^4^ Institute of Translational Pharmacy, Jining Medical Research Academy, Jining, China

**Keywords:** streptozotocin (STZ), diabetes, gas chromatography mass spectrometry (GC-MS), metabolites, metabolomics

## Abstract

**Purpose:**

Type 1 diabetes is characterized by elevated blood glucose levels, which negatively impacts multiple organs and tissues throughout the body, and its prevalence is on the rise. Prior reports primarily investigated the serum and urine specimen from diabetic patients. However, only a few studies examined the overall metabolic profile of diabetic animals or patients. The current systemic investigation will benefit the knowledge of STZ-based type 1 diabetes pathogenesis.

**Methods:**

Male SD rats were arbitrarily separated into control and streptozotocin (STZ)-treated diabetic rats (n = 7). The experimental rats received 50mg/kg STZ intraperitoneal injection daily for 2 consecutive days. Following 6 weeks, metabolites were assessed *via* gas chromatography-mass spectrometry (GC-MS), and multivariate analysis was employed to screen for differentially expressed (DE) metabolites between the induced diabetic and normal rats.

**Results:**

We identified 18, 30, 6, 24, 34, 27, 27 and 12 DE metabolites in the serum, heart, liver, kidney, cortex, renal lipid, hippocampus, and brown fat tissues of STZ-treated diabetic rats, compared to control rats. Based on our analysis, the largest differences were observed in the amino acids (AAs), B-group vitamin, and purine profiles. Using the metabolic pathway analysis, we screened 13 metabolic pathways related to the STZ-exposed diabetes pathogenesis. These pathways were primarily AA metabolism, followed by organic acids, sugars, and lipid metabolism.

**Conclusion:**

Based on our GC-MS analysis, we identified potential metabolic alterations within the STZ-exposed diabetic rats, which may aid in the understanding of diabetes pathogenesis.

## 1 Introduction

Diabetes is an endocrine disease characterized by elevated blood glucose level, which can be divided into type 1 diabetes and type 2 diabetes according to the different pathogenesis. Among them, type 1 diabetes is caused by the destruction of pancreatic beta cells, insufficient insulin secretion, leading to hyperglycemia and even ketosis (1). Studies have shown that there will be approximately 8 million type 1 diabetes patients worldwide in 2021, and the prevalence of type 1 diabetes is predicted to increase by 60-107% to reach 135 million to 174 million in 2040. The substantial missing prevalence highlights the premature mortality from type 1 diabetes. Acute complications include diabetic ketoacidosis, which requires urgent management. Chronic complications include microvascular and macrovascular diseases (2). Type 1 diabetes can occur at any age, and while its onset peak incidence still in early adolescence, it is more common in adulthood, with the majority of incident and prevalent cases occurring in adult population. Macrovascular and microvascular complications are the greatest cause of excess morbidity and mortality in the adult population (3). The increasing global burden of type 1 diabetes, particularly in resource-limited low- and middle-income countries, calls for targeted interventions to optimize blood glucose control to reduce acute and chronic complications (4). Diabetes is also a metabolic disease, affecting the body’s metabolism of matter and energy. It is speculated that the metabolomics findings of diabetes may reveal biological pathways, aid in the understanding of pathophysiological mechanisms, identify individuals at risk of disease, and benefit the development of novel treatments for type 1 diabetes (5).

Genomics and proteomics offer information about what is likely to happen. In contrast, metabolomics provides data on what is actually happening (Bill Lasley, UC Davis). Metabolomics employs high-throughput detection and data analysis to examine the cellular metabolome, which includes all low molecular weight metabolites (molecular weight <1000) within the cell, aims to realize information modeling and system integration in a branch of systems biology. Metabolomics is a research method based on mass spectrometry, and the common separation approaches are as follows: gas chromatography (GC), liquid chromatography (LC), capillary electrophoresis (CE), and so on (6). Metabolomics is a profitable tool for the identification of novel risk markers. Diabetes differential metabolites identification may highlight underlying mechanisms regulating diabetes pathogenesis. Moreover, it can screen patients at risk of disease, which can be a predictive platform for related warning signals (5, 7).

In recent years, there were multiple metabolomics investigations involving diabetic serum and other tissues, such as the heart (8), liver (9), kidney (10), and hippocampus (11). Based on our search of literature, the present article is the first to comprehensively analyze the metabolomics of STZ-treated diabetic rats. Herein, we employed GC-MS, along with univariate and multivariate analyses to elucidate STZ-based alterations in the serum, heart, liver, kidney, cortex, renal lipid, hippocampus, and brown fat metabolites of diabetic rats. Our findings further our understanding of STZ-based diabetes pathogenesis, and may lead to a new perspective on type 1 diabetes treatments and complications management.

## 2 Material and methods

### 2.1 Animals

8 week-old male Sprague-Dawley (SD) rats, averaging 200-230 gram in body weight, were maintained in typical polypropylene cages (three rats/cage), under regulated room temperature (RT) and humidity, with 12/12-hour light-dark cycle. All rats were given ample water as well pelleted regular chow diet for two weeks. Fasting glucose and body weight were measured weekly. Subsequently, the rats were arbitrarily separated into two groups, namely, control (n=7) and STZ-exposed diabetic rats (n=7). Following fasting for 12 hours, each rat in the STZ group was intraperitoneally injected daily with streptozotocin (STZ 50 mg/kg, resuspended in newly prepared 0.1 M, pH 4.5 citrate buffer, and kept on ice prior to usage, low dose STZ model) for two consecutive days to induce diabetes (12). The control rats were provided with the same volume and duration of citrate buffer alone. The STZ dosage was selected based on earlier report (13). In addition, the fasting glucose was measured on the second day after STZ injections to assess the success of the established type 1 diabetes model. Rats with blood glucose ≥11.1mmol/L were considered diabetic. The blood glucose and body weight assessments were performed once a week for 6 consecutive weeks. Our work received ethical approval from the Translational pharmaceutical laboratory, Jining First People’s Hospital (protocol number JNRM-2022-DW-011), and followed the animal care and experimentation guidelines of the National Institutes of Health.

### 2.2 Reagents

STZ, citric acid, and sodium citrate were acquired from Yisheng Biotechnology Co., LTD (Shanghai, China). O-methylhydroxylamine hydrochloride (the purity of 98%) was acquired from J&K Scientific Ltd. (Beijing, China). Heptadecanoic acid (an internal standard, IS, purity ≥98%), N, O-bis (trimethylsilyl) trifluoroacetamide (BSTFA) with 1% trimethyl-chlorosilne (TMCS) were acquired from Sigma-Aldrich (Saint Louis, USA). Pyridine was provided by Macklin Biochemical (Shanghai, China). Chromatographic-grade methanol came from Thermo Fisher Scientific (Waltham, MA,USA). Water was bought from Wahaha Company (Hangzhou, China).

### 2.3 Sample preparation

Following euthanasia, we extracted blood samples and obtained serum samples using centrifugation (4500×*g*, 5 min). Next, 100 μL serum was combined with 350 μL methanol and 100 μg/mL heptadecanoic acid, prior to centrifugation at 20913 × *g* at 4°C for 10 min. The resulting supernatants were placed in 2-mL tubes, prior to drying at 37°C under nitrogen gas, then combined with 80μL O-methylhydroxylamine hydrochloride (resuspended in pyridine at 15 mg/mL), and maintained at 70°C for 90 min. Next, 100 μL BSTFA carrying 1% TMCS was introduced to the extracts, and maintained at 70°C for 60 min. This was followed by vortexing, centrifugation (20913 × *g*, 4°C, 2 min), filter filtration using 0.22-μm membrane filter, and analysis using GC-MS.

Tissue samples (50mg; heart, liver, kidney, cortex, renal lipid, hippocampus, and brown fat) underwent homogenization in 1 methanol and 1 mg/mL IS, prior to transfer to 2-mL tubes and centrifugation at 20913 × *g* at 4°C for 10 min. The rest of the protocol mirrored that of the serum samples.

The quality control (QC) samples were described as a combination of control and STZ-treated diabetic rat samples.

### 2.4 GC-MS analysis

An Agllent 7890B 7000C MSD GC-MS was used to conduct GC-MS analysis. Samples were separated with an HP-5MS fused silica capillary column. Using the carrier helium, 1 μL derivative mixture was operated in split mode (50:1), with flow rates of 3 mL/min front inlet purge and 1 mL/min gas. The temperatures associated with the transfer line, administration, and ion source were 250°C, 280°C, and 230°C, respectively. The GC temperature regimen was as follows: 60°C for 5min, elevation to 300°C, at 8°C/min, then maintenance at 300°C for 5 min. The MS setting employed electron ionization with 20 spectra/s acquisition rate. MS identification was carried out in full scan mode using electrospray ionization (ESI) with m/z range of 50-800.

### 2.5 Multivariate analysis

MassHunter Workstation Unknown Analysis (Agilent, B.06.00) was employed for QC sample data analysis in GC-MS to generate a spectrum library. Next, the MassHunter Quantitative (Agilent, B.06.00) was employed to select the spectrum library to verify compounds.

SMICA-P 14.1 (Sartorius, Sweden) was employed for differential compound analysis. The original GC-MC-based data was preprocessed in Excel with arrangement and area ratio normalization, and the data set was sorted into a pattern that could be directly imported into SIMCA-P software for analysis. The principal component (PCA), partial least squares discriminant (PLS-DA), and orthogonal partial least squares discriminant analyses (OPLS-DA) were employed for model generation, and the model was fitted automatically. The score and load diagrams were viewed for data analysis, and permutation testing (200 permutations) was performed to verify whether the model was over-fitted. To further identify the differential compounds between control and experimental rats, IBM SPSS (Statistical Package for Social Science) version 26.0 (IBM Corp., Armonk, NY, USA) was employed to perform two-tailed Student’s t-tests. Variable importance in projection (VIP) value >1.0 and computed p-value <0.05 were set as the significance threshold.

Pathway assessments were done *via* the MetaboAnalyst 5.0 (https://www.metaboanalyst.ca) and Kyoto Encyclopedia of Genes and Genomes (KEGG; https://www.kegg.jp) analyses. Raw p < 0.05 and impact > 0 were set as significance threshold.

## 3 Results

### 3.1 Assessment of diabetes in STZ-exposed rats

Following two intraperitoneal injections of STZ, the blood glucose of experimental rats was markedly enhanced, relative to control rats, and the body weight showed no gain during the entire experiment (**Table 1**).

**Table 1:** The metabolic parameters of control (CON) and STZ-treated diabetic (STZ) rats.

Parameter	Groups	Weeks of observation			
		week0	week2	week4	week6
Body weight	CON	233.70 ± 3.70	248.19 ± 4.02	265.44 ± 3.61	283.26 ± 4.07
(g)	STZ	231.96 ± 3.36	217.93 ± 2.56*	217.05 ± 4.04*	217.05 ± 3.81*
Blood glucose	CON	5.34 ± 0.06	5.54 ± 0.04	5.51 ± 0.05	5.37 ± 0.07
(mmo/L)	STZ	5.34 ± 0.04	15.89 ± 0.46*	15.14 ± 0.61*	14.94 ± 0.54*

values are presented as mean ± standard error (n=7) in each group, and were analyzed using repeated measures one-way ANOVA in the SPSS v26.0 software (IBM Corp., Armonk, NY, USA). P<0.05 was set as the significance threshold between CON (control) and STZ group. *indicates significant difference (P < 0.05) between CON and STZ group.

### 3.2 Typical GC-MS total ion chromatograms

**Figure 1:**
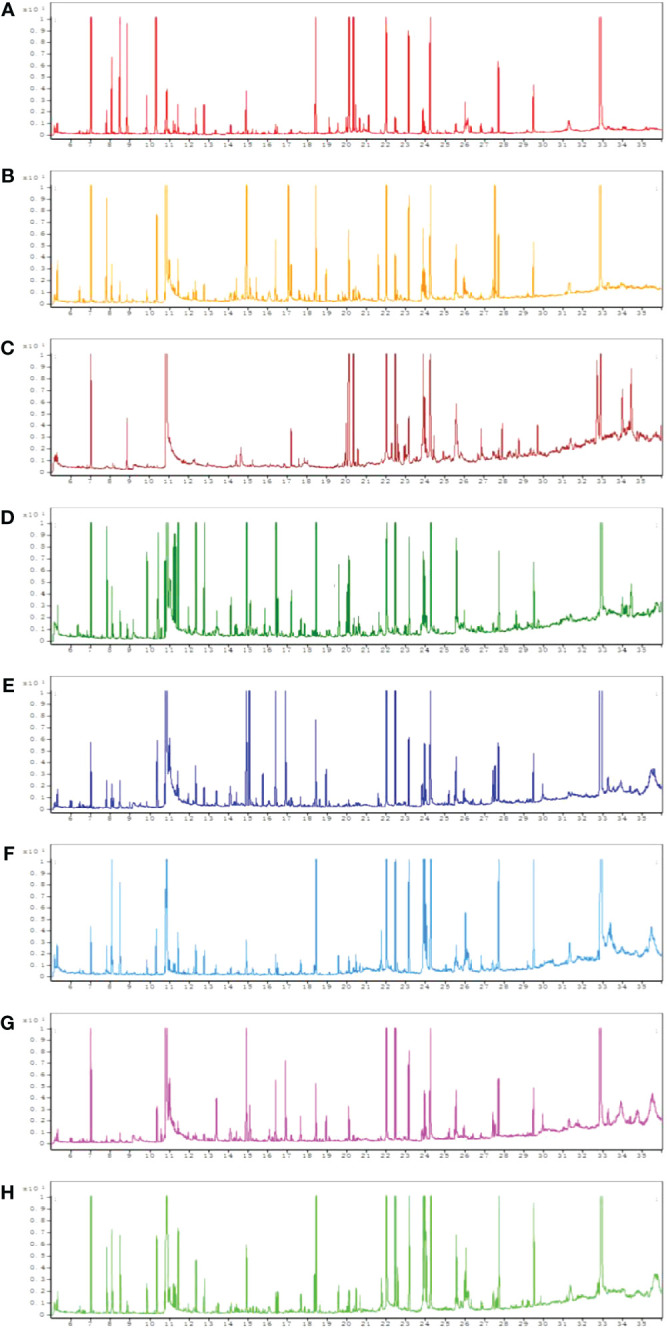
Typical gas chromatography–mass spectrometry (GC–MS) total ion chromatograms (TIC) of the quality control (QC) from serum **(A)**, heart **(B)**, liver **(C)**, kidney **(D)**, cortex **(E)**, renal lipid **(F)**, hippocampus **(G)**, and brown fat **(H)**.

All chromatograms involving the QC serum and tissue samples displayed strong signals. Typical GC-MS total ion chromatograms (TIC) representatives of the serum, heart, liver, kidney, cortex, renal lipid, hippocampus, and brown fat are displayed in **Figure 1**.

### 3.3 Multivariate analysis

The GC-MS results were further verified with the SMICA software, and fitted *via* the OPLS model to identify significant metabolites. IBM SPSS version 26 (IBM Corp., Armonk, NY, USA) was employed for forecasting. Based on our analyses, 18, 30, 6, 24, 34, 27, 27, and 12 metabolites were DE between the control and STZ-treated diabetic rat serum, heart, liver, kidney, cortex, renal lipid, hippocampus, and brown fat tissues, respectively. These parameters (serum: R2X = 0.607, R2Y = 0.981, Q2 = 0.910; heart: R2X = 0.626, R2Y = 0.995, Q2 = 0.906; liver: R2X = 0.554, R2Y = 0.964, Q2 = 0.581; kidney: R2X = 0.734, R2Y = 0.991, Q2 = 0.929; cortex: R2X = 0.739, R2Y = 0.996, Q2 = 0.955; renal lipid: R2X = 0.629, R2Y = 0.993, Q2 = 0.824; hippocampus: R2X = 0.709, R2Y = 0.997, Q2 = 0.969; brown fat: R2X = 0.605, R2Y = 0.996, Q2 = 0.815) clearly separated the control from the STZ-treated rats (VIP > 1, p < 0.05).

**Figure 2:**
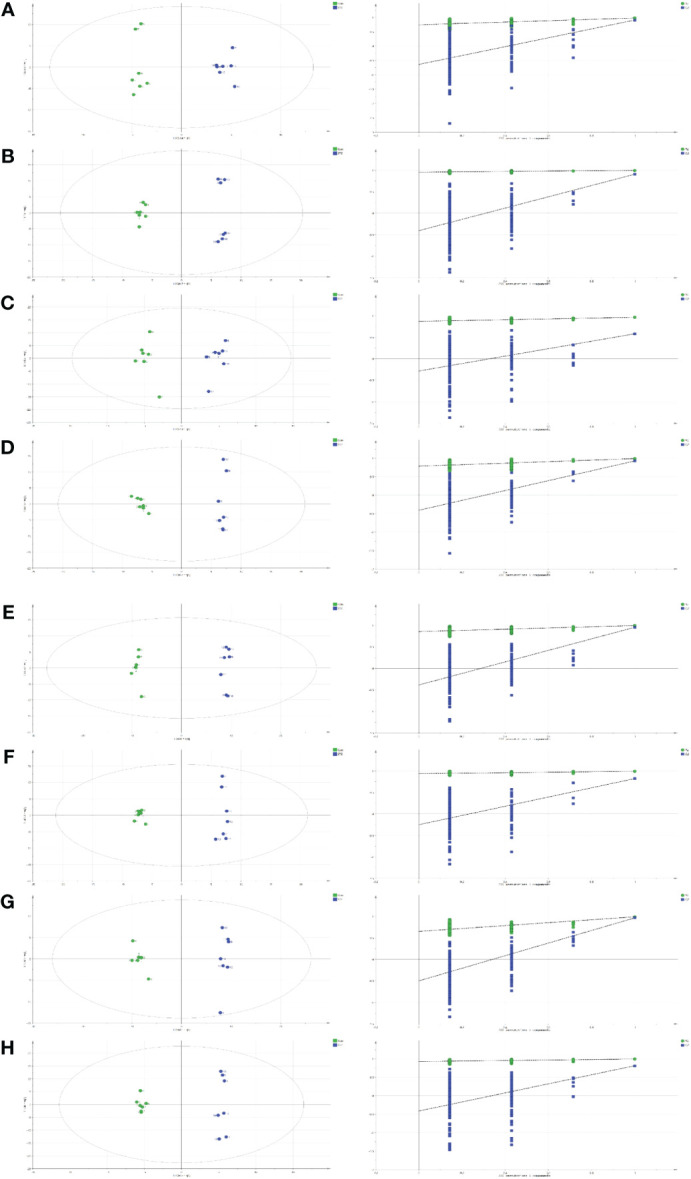
Orthogonal projections to the latent structural (OPLS) scores and 200 permutation tests for the OPLS-discriminant analysis (OPLS-DA) models: serum **(A)**, heart **(B)**, liver **(C)**, kidney **(D)**, cortex **(E)**, renal lipid **(F)**, hippocampus **(G)**, and brown fat **(H)**.

For all parameters, a value closer to 1 represented a better explanatory rate and prediction ability of the overall model. The permutation test carried out sequential replacement of samples, recalculated the statistical test quantity, constructed the empirical distribution, and lastly, computed the OPLS-DA model parameters for judgment. In total, 200 replacement tests were conducted for internal model validation, and to avoid the model from over-fitting. The judgment criterion was as follows: the Q2 intercept on the Y-axis was negative, indicating that over-fitting did not occur (**Figure 2**).

### 3.4 Identification of potential differential metabolites

**Figure 3:**
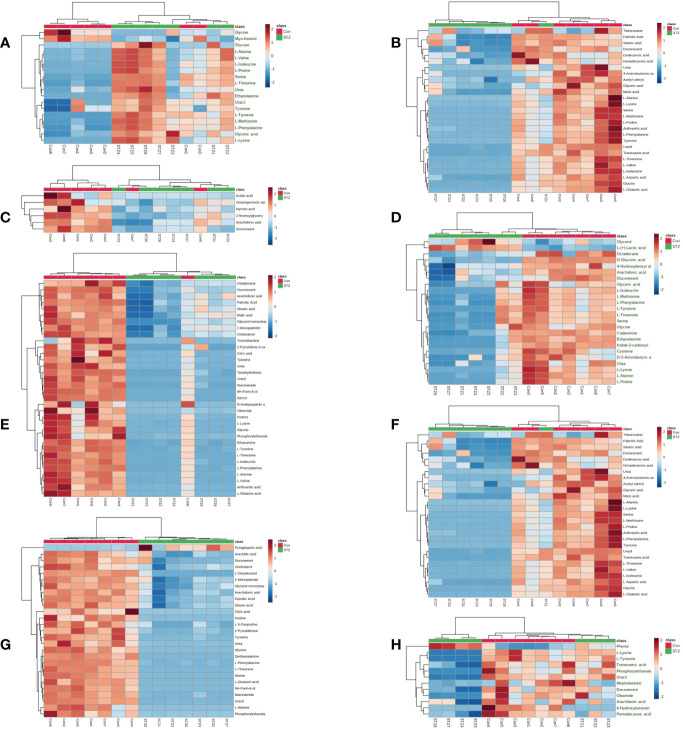
significance (red, elevated; blue, diminished). Rows denote samples, columns denote metabolites.The heatmap of differentially regulated metabolites from the serum **(A)**, heart **(B)**, liver **(C)**, kidney **(D)**, cortex **(E)**, renal lipid **(F)**, hippocampus **(G)**, and brown fat **(H)** of STZ and control rats. The color of each section signifies its alteration.

Using SMICA 14.1 and SPSS 26.0 analyses, VIP > 1.0 and p < 0.05 were set as the significance threshold. The corresponding compounds were considered as differential compounds between the control and STZ-treated rats. There were 18, 30, 7, 24, 34, 27, 27 and 12 DE metabolites between the control and STZ-treated diabetic rat serum, heart, liver, kidney, cortex, renal lipid, hippocampus, and brown fat tissues, respectively (**Table 2**). The relative distributions of all differential metabolites in all tissues are expounded in **Figure 3**.

**Table 2:** ** **A summary of the statistically significant differential metabolites expressed in each tissue of the control and experimental rats.

Metabolites	HMDB	VIP	p-value	Fold Change
Serum				
Ethanolamine	HMDB0000149	1.29	1.31E-03	6.29E+00
Glucose	HMDB0000122	1.20	1.38E-02	5.54E+01
Glyceric acid	HMDB0000139	1.47	4.48E-04	3.37E+00
Glycine	HMDB0000123	1.05	3.82E-02	2.56E-01
L-Alanine	HMDB0000161	1.29	9.99E-04	7.95E+00
L-Isoleucine	HMDB0000172	1.29	1.86E-03	5.75E+00
L-Lysine	HMDB0000182	1.54	4.84E-05	1.17E+01
L-Methionine	HMDB0000696	1.54	4.67E-04	1.17E+01
L-Phenylalanine	HMDB0000159	1.44	5.25E-04	3.98E+00
L-Proline	HMDB0000162	1.32	9.39E-04	7.30E+00
L-Threonine	HMDB0000167	1.31	2.74E-03	3.79E+00
L-Tyrosine	HMDB0000158	1.39	2.42E-03	3.94E+00
L-Valine	HMDB0000883	1.30	9.99E-04	7.95E+00
Myo-Inositol	HMDB0000211	1.52	3.30E-05	4.59E-01
Serine	HMDB0062263	1.52	1.06E-02	3.57E+00
Tyrosine	HMDB0000158	1.16	2.53E-02	1.94E+00
Uracil	HMDB0000300	1.19	1.81E-02	2.09E+00
Urea	HMDB0000294	1.14	1.21E-02	3.96E+00
Heart				
4-Hydroxybutanoic acid	HMDB0000549	1.35	1.00E-03	1.35E+00
9H-Purin-6-ol	HMDB0000157	1.15	4.80E-02	4.63E-01
Arachidonic acid	HMDB0001043	1.04	3.10E-02	7.32E-01
Cholesterol	HMDB0000067	1.22	6.00E-03	7.32E-01
Dulcitol	HMDB0000107	1.11	2.30E-02	1.74E+00
Ethanolamine	HMDB0000149	1.30	3.00E-03	3.13E-01
Glyceric acid	HMDB0000139	1.31	1.00E-03	6.32E-01
Glycine	HMDB0000123	1.16	3.20E-02	4.29E-01
L-5-Oxoproline	HMDB0000267	1.16	1.60E-02	4.84E-01
L-Alanine	HMDB0000161	1.29	6.00E-03	3.48E-01
L-Aspartic acid	HMDB0000191	1.26	4.00E-03	3.20E-01
L-Isoleucine	HMDB0000172	1.24	2.10E-02	4.17E-01
L-Lysine	HMDB0000182	1.29	8.00E-03	3.61E-01
L-Proline	HMDB0000162	1.31	4.00E-03	3.29E-01
L-Threonine	HMDB0000167	1.28	1.10E-02	3.75E-01
L-Tyrosine	HMDB0000158	1.30	6.00E-03	3.74E-01
L-Valine	HMDB0000883	1.21	2.00E-02	3.92E-01
Myo-Inositol	HMDB0000211	1.12	8.00E-03	8.07E-01
Niacinamide	HMDB0001406	1.23	2.10E-02	4.85E-01
Oleamide	HMDB0002117	1.29	2.00E-03	3.08E-01
Palmitic Acid	HMDB0000220	1.15	1.80E-02	7.98E-01
Phosphorylethanolamine	HMDB0000224	1.24	1.20E-02	4.00E-01
Propylamine	HMDB0034006	1.21	2.70E-02	4.63E-01
Putrescine	HMDB0001414	1.11	3.50E-02	4.28E-01
Serine	HMDB0062263	1.11	1.30E-02	3.83E-01
Stearic acid	HMDB0000827	1.25	3.00E-03	6.93E-01
Taurine	HMDB0000251	1.32	2.00E-03	2.78E-01
Trichloroethanol	HMDB0062438	1.32	2.00E-03	2.58E+00
Uracil	HMDB0000300	1.28	5.00E-03	4.23E-01
Urea	HMDB0000294	1.35	1.00E-03	2.76E-01
Liver				
2-linoleoylglycerol	HMDB0011538	1.37	1.70E-02	3.80E-01
Acetic acid	HMDB0000042	1.05	2.70E-02	2.43E-01
Arachidonic acid	HMDB0001043	1.34	3.10E-02	5.67E-01
Doconexent	HMDB0002183	1.34	1.80E-02	2.67E-01
Myristic acid	HMDB0000806	1.39	3.80E-02	5.40E-01
Octaneperoxoic acid, 1,1-dimethylethyl ester	HMDB0032827	1.28	3.70E-02	4.87E-01
Kidney				
4-Hydroxybenzyl alcohol	HMDB0011724	1.24	3.00E-03	5.25E-01
Arachidonic acid	HMDB0001043	1.31	3.00E-03	4.77E-01
Cadaverine	HMDB0002322	1.33	1.00E-03	2.95E-01
Cysteine	HMDB0000574	1.28	1.00E-03	1.88E-01
D-2-Aminobutyric acid	HMDB0000650	1.12	1.00E-03	3.74E-01
D-Gluconic acid	HMDB0000625	1.52	7.73E-11	2.10E-01
Doconexent	HMDB0002183	1.23	1.00E-03	4.22E-01
Ethanolamine	HMDB0000149	1.34	1.61E-04	1.96E-01
Glyceric acid	HMDB0000139	1.02	2.90E-02	4.48E-01
Glycerol	HMDB0000131	1.10	2.70E-02	1.29E+00
Glycine	HMDB0000123	1.12	1.30E-02	3.27E-01
Indole-2-carboxylic acid	HMDB0002285	1.41	7.00E-03	1.62E-01
L-(+)-Lactic acid	HMDB0000190	1.49	1.20E-07	1.72E+00
L-Alanine	HMDB0000161	1.42	4.20E-05	1.47E-01
L-Isoleucine	HMDB0000172	1.33	4.90E-04	2.06E-01
L-Lysine	HMDB0000182	1.37	2.03E-03	1.20E-01
L-Methionine	HMDB0000696	1.28	2.00E-06	2.82E-01
L-Phenylalanine	HMDB0000159	1.24	5.00E-03	3.19E-01
L-Proline	HMDB0000162	1.40	1.03E-04	1.60E-01
L-Threonine	HMDB0000167	1.21	1.00E-02	3.22E-01
L-Tyrosine	HMDB0000158	1.12	2.30E-02	3.41E-01
Octadecane	HMDB0033721	1.10	7.00E-03	3.13E-01
Serine	HMDB0000187	1.16	1.80E-02	3.35E-01
Urea	HMDB0000294	1.29	1.00E-03	2.85E-01
Cortex				
1-Monopalmitin	HMDB0011564	1.14	4.63E-04	3.35E-01
2-Pyrrolidone-5-carboxylic acid	HMDB0000267	1.07	1.00E-03	1.44E-01
9H-Purin-6-ol	HMDB0000157	1.25	4.70E-05	1.09E-03
Anthranilic acid	HMDB0001123	1.18	4.78E-04	5.32E-03
Arachidonic acid	HMDB0001043	1.09	1.00E-03	3.75E-01
Cholesterol	HMDB0000067	1.16	2.32E-02	2.80E-01
Citric acid	HMDB0000094	1.19	3.13E-04	1.83E-02
Doconexent	HMDB0002183	1.22	5.00E-03	2.49E-01
Ethanolamine	HMDB0000149	1.27	5.00E-06	1.92E-03
Glycerol monostearate	HMDB0011535	1.20	1.50E-05	1.85E-01
Glycine	HMDB0000123	1.20	2.32E-04	1.08E-03
Inosine	HMDB0000195	1.14	1.00E-03	6.48E-03
L-Alanine	HMDB0000161	1.22	1.20E-04	1.13E-03
L-Glutamic acid	HMDB0000148	1.19	4.32E-04	1.34E-04
L-Isoleucine	HMDB0000172	1.25	4.30E-05	2.40E-02
L-Lysine	HMDB0000182	1.18	7.00E-06	2.40E-02
L-Phenylalanine	HMDB0000159	1.26	1.20E-05	1.47E-02
L-Threonine	HMDB0000167	1.26	1.90E-05	3.56E-03
L-Tyrosine	HMDB0000158	1.25	2.20E-05	1.57E-02
L-Valine	HMDB0000883	1.23	8.50E-05	4.27E-03
Malic acid	HMDB0000744	1.09	4.66E-08	3.74E-01
N-Acetylaspartic acid	HMDB0000812	1.02	3.00E-03	2.79E-03
Niacinamide	HMDB0001406	1.26	9.54E-07	2.20E-02
Octadecane	HMDB0033721	1.26	2.00E-03	4.00E-01
Oleamide	HMDB0002117	1.07	5.00E-03	3.08E-02
Palmitic Acid	HMDB0000220	1.17	1.46E-04	3.61E-01
Phosphorylethanolamine	HMDB0000224	1.12	1.00E-03	3.20E-02
Serine	HMDB0000187	1.26	2.40E-05	1.02E-03
Stearic acid	HMDB0000827	1.13	1.00E-03	3.71E-01
Tetrahydrofuran	HMDB0000246	1.25	3.08E-08	3.71E-02
Tromethamine	HMDB0240288	1.12	3.06E-04	2.12E-03
Tyrosine	HMDB0000158	1.17	1.00E-03	2.03E-02
Uracil	HMDB0000300	1.24	5.80E-05	1.58E-02
Urea	HMDB0000294	1.21	2.12E-04	1.58E-02
Renal Lipid				
4-Aminobutanoic acid	HMDB0000112	1.17	4.00E-03	1.53E-01
Acetyl valeryl	HMDB0031476	1.16	2.00E-02	5.67E-01
Anthranilic acid	HMDB0001123	1.30	1.67E-04	9.53E-02
Doconexent	HMDB0002183	1.05	6.00E-03	3.84E-01
Dodecanoic acid	HMDB0000638	1.02	1.40E-02	3.42E-01
Ethanolamine	HMDB0000149	1.32	1.81E-04	3.42E-01
Glyceric acid	HMDB0000139	1.09	7.00E-03	2.88E-01
Glycine	HMDB0000123	1.30	1.00E-03	1.29E-01
L-Alanine	HMDB0000161	1.29	1.00E-03	2.13E-02
L-Aspartic acid	HMDB0000191	1.32	1.99E-04	1.56E-01
L-Glutamic acid	HMDB0000148	1.33	1.36E-04	9.14E-02
L-Isoleucine	HMDB0000172	1.32	1.63E-03	9.40E-02
L-Lysine	HMDB0000182	1.33	3.70E-05	3.24E-02
L-Methionine	HMDB0000696	1.36	2.12E-04	5.68E-02
L-Phenylalanine	HMDB0000159	1.35	2.80E-05	5.30E-02
L-Proline	HMDB0000162	1.32	1.00E-04	7.22E-02
L-Threonine	HMDB0000167	1.33	1.22E-04	1.02E-01
L-Valine	HMDB0000883	1.30	2.50E-02	9.64E-02
Malic acid	HMDB0000744	1.22	3.00E-03	2.72E-01
Nonadecanoic acid	HMDB0000772	1.02	7.00E-03	4.18E-01
Palmitic Acid	HMDB0000220	1.17	1.80E-02	4.13E-01
Serine	HMDB0062263	1.36	3.20E-05	6.20E-02
Stearic acid	HMDB0000827	1.16	2.30E-02	4.06E-01
Tranexamic acid	HMDB0014447	1.28	3.78E-04	1.85E-01
Tyrosine	HMDB0000158	1.34	4.70E-05	3.92E-02
Uracil	HMDB0000300	1.30	1.00E-03	1.70E-01
Urea	HMDB0000294	1.28	2.00E-03	1.13E-02
Hippocampus				
1-Monopalmitin	HMDB0011564	1.20	4.20E-05	3.61E-01
1-Octadecanol	HMDB0002350	1.26	1.53E-08	9.16E-02
2-Pyrrolidinone	HMDB0002039	1.22	9.50E-05	5.78E-02
9H-Purin-6-ol	HMDB0000157	1.27	7.00E-06	7.29E-04
Arachidic acid	HMDB0002212	1.14	3.88E-04	3.11E-01
Arachidonic acid	HMDB0001043	1.23	7.00E-06	3.87E-01
Cholesterol	HMDB0000067	1.17	1.39E-04	3.71E-01
Citric acid	HMDB0000094	1.08	2.00E-03	8.85E-03
Diethanolamine	HMDB0004437	1.28	3.89E-07	2.30E-04
Doconexent	HMDB0002183	1.13	2.57E-04	3.53E-01
Glycerol monostearate	HMDB0011535	1.22	4.00E-06	3.00E-01
Glycine	HMDB0000123	1.25	3.20E-05	4.03E-04
Inosine	HMDB0000195	1.20	1.84E-04	1.23E-02
L-5-Oxoproline	HMDB0000267	1.19	1.70E-05	1.22E-01
L-Alanine	HMDB0000161	1.19	3.35E-04	1.17E-03
L-Glutamic acid	HMDB0000148	1.27	1.00E-05	1.40E-04
L-Phenylalanine	HMDB0000159	1.28	7.86E-07	2.57E-02
L-Threonine	HMDB0000167	1.28	3.00E-06	1.26E-03
Niacinamide	HMDB0001406	1.28	4.55E-10	3.68E-02
Palmitic Acid	HMDB0000220	1.25	3.08E-07	3.23E-01
Phosphorylethanolamine	HMDB0000224	1.13	2.00E-03	1.30E-02
Pyroglutamic acid	HMDB0000267	1.06	1.00E-02	2.45E+01
Serine	HMDB0062263	1.28	4.00E-06	1.25E-03
Stearic acid	HMDB0000827	1.22	7.00E-06	3.62E-01
Tyrosine	HMDB0000158	1.24	4.90E-05	2.22E-02
Uracil	HMDB0000300	1.29	1.57E-07	1.34E-02
Urea	HMDB0000294	1.21	2.24E-04	6.90E-03
Brown Fat				
4-Hydroxybutanoic acid	HMDB0000549	1.56	4.00E-03	4.27E-02
Arachidonic acid	HMDB0001043	1.16	3.40E-02	5.90E-01
Doconexent	HMDB0002183	1.33	1.60E-02	4.62E-01
L-Lysine	HMDB0000182	1.27	3.20E-02	4.29E-01
L-Tyrosine	HMDB0000158	1.28	3.10E-02	4.23E-01
Mephobarbital	HMDB0014987	1.15	1.70E-02	4.23E-01
Oleamide	HMDB0002117	1.20	2.80E-02	3.72E-01
Pentadecanoic acid	HMDB0000826	1.53	1.00E-03	4.52E-01
Phenol	HMDB0000228	1.58	2.71E-04	4.20E+00
Phosphorylethanolamine	HMDB0000224	1.58	3.80E-02	4.17E-01
Tranexamic acid	HMDB0014447	1.22	3.50E-02	5.31E-01
Uracil	HMDB0000300	1.29	4.60E-02	4.60E-01

### 3.5 Analysis of metabolic axes

**Figure 4:**
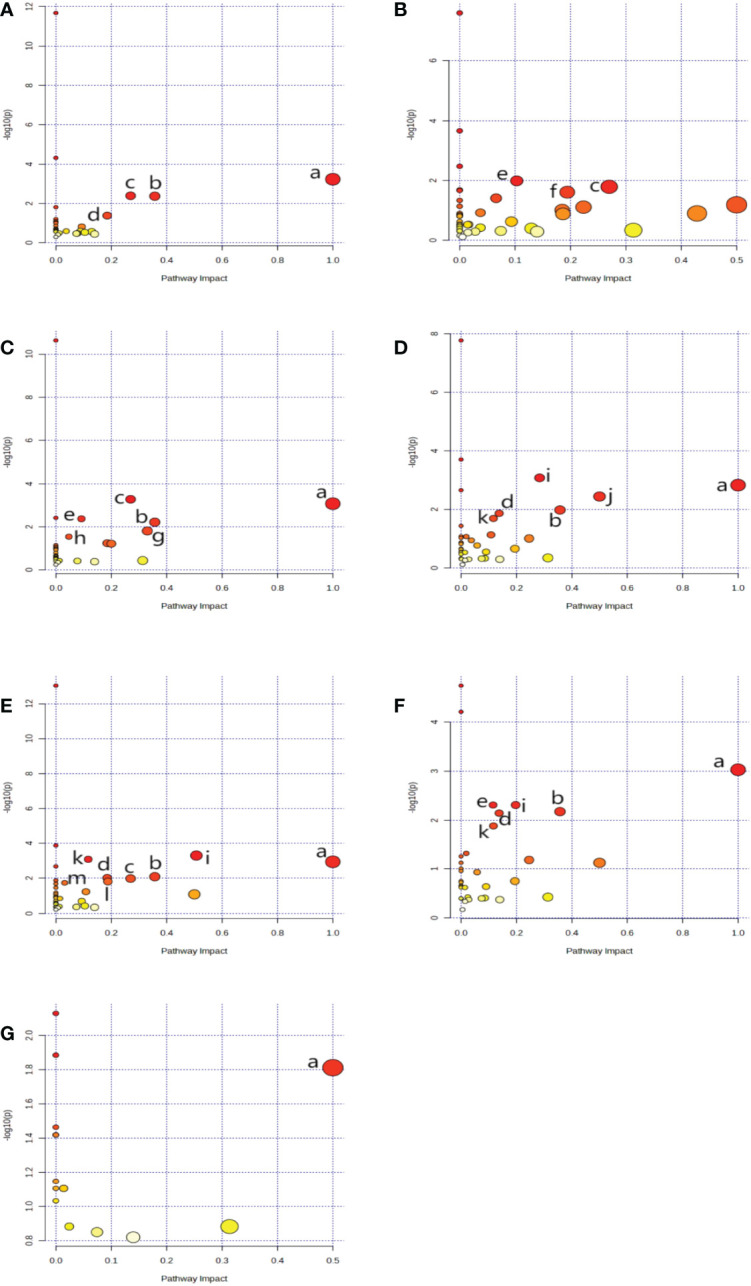
** **A network analysis summary, as evidenced by MetaboAnalyst 5.0. Serum **(A)**, Heart **(B)**, kidney **(C)**, cortex **(D)**, renal lipid **(E)**, hippocampus **(F)**, and brown fat **(G)**. Metabolisms or biosynthesis of (a) Phenylalanine, tyrosine, and tryptophan, (b) Phenylalanine, (c) Glycine, serine, and threonine, (d) Glyoxylate and dicarboxylate, (e) Glutathione (f) Nicotinate and nicotinamide, (g) Glycerolipid, (h) Pentose phosphate, (i) Alanine, aspartate, and glutamate, (j) D-Glutamine and D-glutamate, (k) Arginine, (l) Arginine and proline, (m) Butanoate.

**Figure 5:**
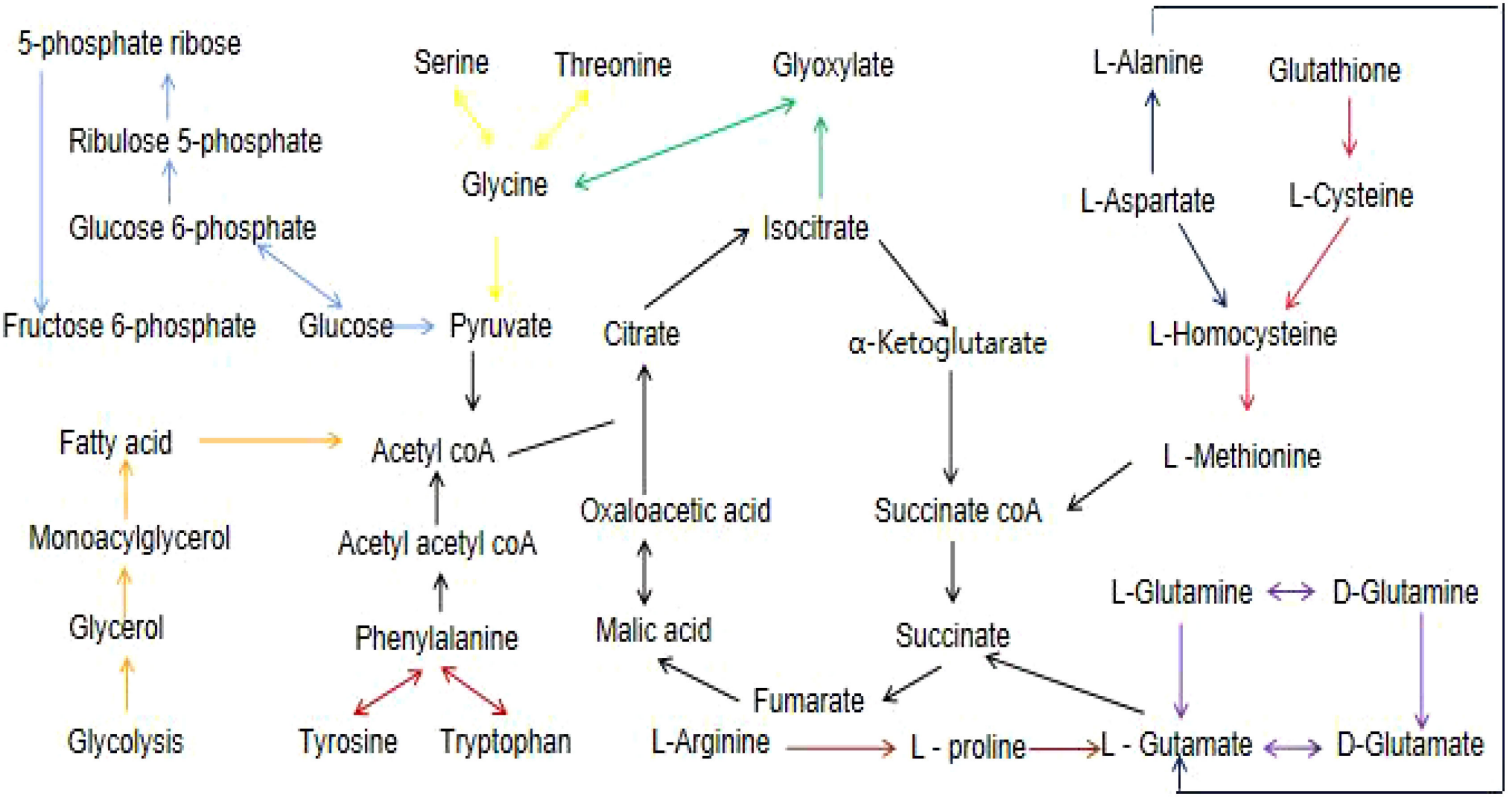
** **A flow chart of the metabolites and their associated networks in the target tissues of STZ-treated diabetic rats versus controls. Red arrows: phenylalanine, tyrosine and tryptophan biosynthesis; light blue arrows: pentose phosphate pathway; yellow arrows: glycine,serine, and threonine metabolism; green arrows: glyoxylate and dicarboxylate metabolism; blue arrows: alanine, aspartate, and glutamate metabolism; pink arrows: glutathione metabolism; purple arrows: D-glutamine and D-glutamate metabolism; orange arrows: glycerolipid metabolism; brown arrows: arginine and proline metabolism.

Using metaboAnalyst 5.0, we identified 13 major pathways that were modulated by the DE metabolites (Raw p < 0.05, impact > 0). In the serum, they were phenylalanine (Phe), tyrosine (Tyr) and tryptophan (Trp) biosynthesis; Phe metabolism; glycine (Gly), serine (Ser), and threonine (Thr) metabolism; as well as glyoxylate (Glyox) and dicarboxylate (DIC) metabolism. In the heart, they were glutathione (GSH) metabolism; Gly, Ser, and Thr metabolism; nicotinate and nicotinamide (NAM) metabolism. In the kidney, they were Gly, Ser, and Thr metabolism; Phe, Tyr, and Trp biosynthesis; GSH, Phe, glycerolipid (GL) metabolism; and pentose phosphate pathway(PPP). In the cortex, they were alanine (Ala), aspartate (Asp) and glutamate (Glu) metabolism; Phe, Tyr, and Trp biosynthesis; D-glutamine and D-glutamate metabolism; Phe, Glyox and DIC metabolism; and arginine (Arg) biosynthesis. In the renal lipid, they were Ala, Asp, and Glu metabolism; Arg, Phe, Tyr, and Trp biosynthesis; Phe metabolism; Glyox and DIC metabolism; Gly, Ser, and Thr, Arg and proline (Pro), and butanoate metabolism. In the hippocampus, they were Phe, Tyr, and Trp biosynthesis, Phe metabolism; Ala, Asp, Glu, Glyox, DIC, and GSH metabolism; and Arg biosynthesis.In brown fat, they were Phe, Tyr, and Trp biosynthesis. The network analyses are detailed in **Table 3** and **Figure 4**, and the information is summarized in **Figure 5**. Furthermore, all network flowcharts are available at KEGG.

**Table 3:** List of metabolic pathways, as evidenced by MetaboAnalyst5.0.

Pathway Name	Raw p	Impact
Serum		
Phenylalanine, tyrosine and tryptophan biosynthesis	6.71E-04	1.00E+00
Phenylalanine metabolism	4.84E-03	3.57E-01
Glycine, serine and threonine metabolism	4.88E-03	2.70E-01
Glyoxylate and dicarboxylate metabolism	4.63E-02	1.85E-01
Heart		
Glutathione metabolism	1.41E-02	1.03E-01
Glycine, serine and threonine metabolism	2.20E-02	2.70E-01
Nicotinate and nicotinamide metabolism	3.05E-02	1.94E-01
Kidney		
Glycine, serine and threonine metabolism	9.52E-04	2.70E-01
Phenylalanine, tyrosine and tryptophan biosynthesis	1.13E-03	1.00E+00
Glutathione metabolism	6.46E-03	9.22E-02
Phenylalanine metabolism	8.08E-03	3.57E-01
Glycerolipid metabolism	2.05E-02	3.30E-01
Pentose phosphate pathway	3.74E-02	4.71E-02
Cortex		
Alanine, aspartate and glutamate metabolism	1.49E-03	2.84E-01
Phenylalanine, tyrosine and tryptophan biosynthesis	1.98E-03	1.00E+00
D-Glutamine and D-glutamate metabolism	4.84E-03	5.00E-01
Phenylalanine metabolism	1.39E-02	3.57E-01
Glyoxylate and dicarboxylate metabolism	2.03E-02	1.38E-01
Arginine biosynthesis	2.68E-02	1.17E-01
Renal Lipid		
Alanine, aspartate and glutamate metabolism	9.71E-04	5.07E-01
Arginine biosynthesis	1.35E-03	1.17E-01
Phenylalanine, tyrosine and tryptophan biosynthesis	1.59E-03	1.00E+00
Phenylalanine metabolism	1.12E-02	3.57E-01
Glyoxylate and dicarboxylate metabolism	1.51E-02	1.85E-01
Glycine, serine and threonine metabolism	1.64E-02	2.70E-01
Arginine and proline metabolism	2.40E-02	1.88E-01
Butanoate metabolism	2.49E-02	3.18E-02
Hippocampus		
Phenylalanine, tyrosine and tryptophan biosynthesis	1.35E-03	1.00E+00
Phenylalanine metabolism	9.59E-03	3.57E-01
Alanine, aspartate and glutamate metabolism	8.29E-03	1.97E-01
Glyoxylate and dicarboxylate metabolism	1.21E-02	1.38E-01
Glutathione metabolism	8.29E-03	1.15E-01
Arginine biosynthesis	1.87E-02	1.17E-01
Brown Fat		
Phenylalanine, tyrosine and tryptophan biosynthesis.	3.06E-02	5.00E-01

## 4 Discussion

There is an almost complete lack of insulin in type 1 diabetes (14), which acts as an anabolic hormone with multiple effects on sugar, lipid, protein metabolism, growth and development. Patients with type 1 diabetes need to use insulin throughout their life to maintain life, and even regular use of insulin cannot simulate the physiological endogenous insulin secretion pattern (15), resulting in large fluctuations in blood sugar, enhanced health care costs (16), and reduced quality of life (17). STZ is an antitumor antibiotic produced from a certain type of streobasidium pullulans. It possesses a targeted destructive effect on pancreatic β cells, and it compromises insulin production to induce diabetes (18). Different dosages of STZ is known to induce different models of diabetes (19). Metabolomics involves an extensive analysis of metabolites within biological systems, namely, low-molecular weight biochemical compounds like sugars, lipids, amino acids (AAs), organic acids, and nucleotides (20). In this study, STZ-treated type 1 diabetic rats were employed for the analysis of compounds with varying performances in the serum and key target tissues of control and experimental rats, under conditions of hyperglycemia. Our goal was to better understand the type 1 diabetes pathogenesis in order to develop novel targets that prevent and/or treat type 1 diabetes and its complications.

As seen in **Table 1**, type 1 diabetic rat models were generated *via* two intraperitoneal injections of STZ (50mg/kg) in ice-cold citrate buffer (pH7.4). Roche blood glucose meter was used to monitor blood glucose weekly, and a blood glucose value greater than 11.1mmol/L was considered to indicate diabetes. The above data revealed that the rat models were successfully established.

We are the first to report an analysis of metabolic alterations in the serum, heart, liver, kidney, cortex, renal lipid, hippocampus, and brown fat of STZ-induced type 1 diabetic rats versus controls. This study revealed that, compared to controls, there were 18, 30, 6, 24, 34, 27, 27, and 12 DE compounds in the serum, heart, liver, kidney, cortex, renal lipid, hippocampus, and brown adipocytes of STZ rats, respectively. These compounds are related to one another, and they participate in metabolic networks associated with sugars, AAs, and energy metabolism. Based on our analysis of these DE compounds and related pathways, we gained a deeper understanding of the metabolic alterations caused by type 1 diabetes. These metabolites and metabolic networks are potential candidate for the indication of early alterations with diabetes, which may enable us to comprehensively understand type 1 diabetes pathogenesis, and provide new targets for its management.

Our analyses uncovered that AAs serve critical functions in STZ-treated type 1 diabetes conditions. Herein, a variety of AA metabolites were DE in the STZ-treated diabetes rats, relative to the controls. These included Cysteine (Cys), Gly, L-5-Oxoproline, L-Ala, L-Asp, L-Glu, L-Isoleucine (Ile), L-Lysine, L-Methionine (Met), L-Phe, L-Proline (Pro), L-Thr, L-Tyr, L-Valine (Val), Pyroglutamic acid, Ser, and Tyr. The associated metabolic networks were as follows: Ala, Asp, Glu, Arg, Pro, Arg, D-glutamine, D-glutamate, Gly, Ser, Thr, Phe, Tyr, and Trp biosynthesis.

Type 1 diabetes occurs in the absence or insufficiency of insulin secretion (21). This often accompanies oxidative stress and alterations in the glucose and lipid metabolism (22–24). Branched-chain AAs (BCAAs) include Leucine (Leu), Ile, and Val, as well as aromatic AAs (AAAs) like Phe, Tyr, and Trp. Previous studies suggested that the serum concentration of BCAAs and AAAs are related to insulin resistance (25–28), and that they can predict future type 2 diabetes development (29). In addition to adverse effects on insulin sensitivity, elevated plasma BCAAs stimulate insulin secretion, deplete insulin reserves in early type 1 diabetes, impair pancreatic β cell function, and ultimately leads to insulin deficiency (30). The levels of BCAAs are also elevated in model animals with type 1 diabetes. The mechanism may be that in the state of insulin deficiency, the amination of BCAAs in the visceral tissue liver is activated, the absorption of BCAAs in the muscle tissue is reduced, the hydrolysis of total muscle protein is increased, the concentration of BCAAs is increased (31). In this study, the levels of BCAAs (Ile, Val) and AAAs (Phe, Tyr) in the serum of STZ rats were markedly elevated, compared to controls, thus corroborating the data from previous investigations. Some investigations also revealed that alterations in BCAAs and AAAs levels also precede increases in blood sugar (32).

Gluconeogenesis is essential for glucose homeostasis (33). We observed elevated serum glycolic AAs (Ala, Met, Pro, Val, and Ser) levels in STZ-treated diabetic rats, which may induce hyperglycemia *via* hepatic or renal gluconeogenesis. Meanwhile, the circulating ketogenic AAs (Lys) and ketogenic and glycolic AAs (lle, Phe, Thr, and Tyr) concentrations increased, suggesting that the body weight of STZ rats was lower, compared to the control rats likely due to insufficient energy synthesis, reduced anabolism, and enhanced catabolism during diabetes. As previously revealed, Ile, Phe, Tyr, and Val all have “dual” identities, which further affect Phe, Tyr, and Trp biosynthesis, as well as Gly, Ser, and Thr metabolic networks. This causes a series of metabolic alterations mediated by diabetes. Our study supported the relationship between AA concentrations and diabetes risk observed in recent years (32, 34), and it facilitated the identification of a potential mechanism to explain this relationship in the future. Alterations in Ala concentration, observed in our study, were related to the metabolic pathways of Glyox and DIC. Using the Glyox cycle, fats are converted to sugars, and the resulting DIC is used as a supplement for compounds in the tricarboxylic acid (TCA) cycle, thereby affecting energy metabolism (4). Elevated Glyox levels may be a new metabolic feature of type 1 diabetes mellitus and its pathophysiology. These findings may help in treating STZ-induced type 1 diabetes.

Gly, one of the simplest AAs and a substrate for GSH biosynthesis, is known to enhance antioxidant defenses. Our study observed a decrease in Gly levels in the serum, heart, kidney, cortex, renal lipids, and hippocampus of STZ-treated type 1 diabetic rats, suggesting that the antioxidant defenses were weakened when the blood glucose was elevated (35). Asp serves as a carrier for K^+^ and Mg^2+^ ions, and it delivers these electrolytes to the myocardium to improve myocardial systolic activity, while lowering oxygen consumption. This has a protective effect on the myocardium, particularly during hypoxia. A previous study revealed that Asp protects the heart (36). A decrease in the cardiac Asp levels in experimental rats suggests that the protective mechanism of the myocardia was affected in presence of elevated blood glucose. Glu provides energy to the brain tissue, and improves the maintenance of brain function (37). The levels of L-Glu in the hippocampal tissue of experimental rats diminished, suggesting that the energy metabolism of the brain was inhibited during hyperglycemia. In addition, a recent study revealed that the levels of plasma Glu also dropped significantly in people with type 1 diabetes (38). Taurine, a sulfurated amino acid derivative, alleviates hyperglycemia induced by STZ in type 1 diabetic mice, inhibits oxidative stress, and improves diabetes and its complications by upregulating glucose transporter (GLUT-2) expression (39). The reduction of taurine levels in the heart of STZ rats will lead to further studies on what role taurine plays.

Type 1 diabetes exhibits mild dyslipidemia (40), and vascular sclerosis (41). Changes in lipid metabolism have important effects on cell function, metabolism, inflammation and oxidative stress (42). Cardiovascular disease is the leading cause of morbidity and mortality in patients with type 1 diabetes (43). Free fatty acids (FFAs) have been concerned to replace glucose as the main energy source for the heart of patients with type 1 diabetes (44). FFAs are typically categorized as either saturated or unsaturated FFA. Unsaturated FFA are further separated into mono- and polyunsaturated FA (PUFA). Based on our analysis, relative to the control, no FFA alterations were detected in the serum of STZ-treated rats. However, the contents of PUFA arachidonic (ARA) and docosahexaenoic acids (DHA) were reduced in the heart, liver, kidney, cortex, renal lipid, hippocampus, and brown fat of STZ-treated rats. DHA is an omega-3 essential fatty acid, which is essential for brain structure and activity maintenance (45), and it is known to reduce the risk of heart disease (46). ARA is a PUFA that serves as a substrate for a range of bioactive compounds synthesis, such as, prostaglandins, thrombutane, and leukotrienes (47). Many brain disorders, such as, Alzheimer’s disease and bipolar disorder (48, 49), appear to be related to PUFA metabolic disorders. The levels of ARA and DHA were reduced in the target organs of diabetic rats. The lipotoxicity hypothesis is widely accepted (50), and it may be related to the involvement of saturated FA. In this study, the levels of palmitic, stearic(also known as octadecanoic acid or C18:0), arachidonic, myristic (also known as tetracanoic acid or C14:0), and pentacarboxylic in the heart, liver, cortex, kidney lipid, hippocampus, and brown adipose tissue of STZ-treated diabetic rats were markedly diminished, compared to controls. Other lipids 1-monoflavin-MG (0:0/18:2(9Z,12Z)/0:0) and glycerin monostearate MG (0:0/18:0/0:0) in the above target tissues were also reduced, relative to controls. The serum exhibited no abnormality of lipid metabolism. It is speculated that in the early stages of type 1 diabetes, each key target organ actively self-regulates to maintain a normal lipid metabolism in the blood. Alterations in these tissue metabolites occur earlier than the detectable alterations in the blood. Thus, the tissue metabolites hold great potential as candidate differential metabolites for predicting type 1 diabetes-related vascular complications.

The TCA or citric acid cycle (51), is a robust biological system of producing energy *via* sugar or other substances oxidization. It is the final metabolic network of the three nutrients (sugars, lipids and AAs), and the hub of metabolic interactions among sugars, lipids, AAs, nucleic acids, and energy metabolism. Type 1 diabetes is often associated with energy metabolism disorders. In this study, we also revealed that the serum Glu levels in STZ rats were elevated, the citric acid levels in the cortex and hippocampus were diminished, and the lactic acid content in the kidney was increased, compared to controls. This likely involved the pentose phosphate pathway and Ala-Asp-Glu metabolism. The aforementioned data suggested that, when insulin deficiency causes hyperglycemia, glucose decomposition and utilization become impaired, Ala is transformed into pyruvate and glutamic acid *via* joint deamination in the body, and pyruvate is acted upon by pyruvate dehydrogenase complex to generate acetyl coenzyme A, which enters the TCA cycle and generates ATP to energize the body.

Glu, Gly, and Cys combine to form GSH (52). GSH metabolism interacts with toxins or drugs to eliminate their toxic effects from the body, and being a strong reducing agent, GSH also participates in a variety of REDOX reactions *in vivo*. In STZ rats, we demonstrated diminished contents of Glu in the cortex, renal lipid, and hippocampus; Gly in the serum, heart, kidney, cortex, renal lipid, hippocampus; and cysteine in the kidney. The raw materials for GSH synthesis were thus reduced, therefore diminishing the antioxidant capacity of the body.

In addition, we also observed marked decreases in the levels of hypoxanthine (9h-Purin-6-ol), hypoxanthosine(inosine), and uracil in the heart, kidney, cortex, renal lipid, hippocampus, and brown adipocytes of STZ rats. These metabolites are strongly correlated with nucleotide synthesis and inflammation prevention. Thus, hypoxanthine, hypoxanthosine, and Ura are excellent candidates as indicators of early type 1 diabetes.

Among the limitations of our research are the following: we only employed a singular metabolomics platform, GC-MS. To better understand type 1 diabetes pathogenesis, additional investigations, based on a combination of metabolomics, proteomics, and transcriptomics, are required to validate our findings. In addition, it is critical to elucidate the effect of STZ on other tissues, such as, lungs, spleen, stomach, pancreas, skin, bladder, and nerves, to fully understand the mechanisms of STZ-induced diabetes.

## 5 Conclusion

Using GC-MS analysis, we extensively evaluated the metabolic alterations occurring within STZ-treated type 1 diabetic rat serum, heart, liver, kidney, cortex, renal lipid, hippocampus, and brown fat. We observed marked alterations related to the AAs, sugars, lipids, and energy metabolism in the STZ-treated rats versus controls. Overall, our data provided the first systematic analysis of STZ-induced type 1 diabetes, which may aid in the enhanced comprehension of STZ-induced type 1 diabetes and related complications pathogenesis. Type 1 diabetes has multifactorial etiology, and it demands further investigations based on the metabolic examinations in humans, animals, and cells.

## Data availability statement

The original contributions presented in the study are included in the article/supplementary material. Further inquiries can be directed to the corresponding authors.

## Ethics statement

The animal study was reviewed and approved by the Ethics Committeee of Jining First People’s Hospital (No. JNRM-2022-DW-011, Jining, China).

## Author contributions

All authors listed have made a substantial, direct and intellectual contribution to the work, and approved it for publication.
